# The impact of attitude toward COVID-19 vaccine on intention to receive influenza vaccination: a multi-group comparison based on the influence of presumed influence model and spillover effects

**DOI:** 10.3389/fpubh.2024.1398680

**Published:** 2024-08-21

**Authors:** Yun Zhang, Hongfa Yi

**Affiliations:** School of Journalism and Communication, Shanghai University, Shanghai, China

**Keywords:** influence of presumed influence model, spillover effects, influenza vaccine, multi-group comparison, COVID-19

## Abstract

**Introduction:**

Influenza vaccination is one of the most important strategies for preventing influenza. However, the influenza vaccination rate in China remains low. During the COVID-19 pandemic, people held different attitudes toward the COVID-19 vaccine. In the post-pandemic era, do the varying attitudes toward the COVID-19 vaccine affect the intention to receive influenza vaccination?

**Methods:**

Based on the influence of presumed influence (IPI) model and spillover effects, this study employed structural equation modeling for multi-group comparison to analyze questionnaires from 613 participants, using instruments such as the Perceived Media Influence on Others Scale (PMIO), the Susceptibility to Influenza Scale (SI), and the Attitude toward Influenza Vaccine Scale (AIV).

**Results:**

The key findings are as follows: (1) Information exposure to the influenza vaccine significantly influences perceived media influence on others. (2) Perceived media influence on others does not directly impact the intention to receive influenza vaccination but rather affects it through attitude toward the influenza vaccine. (3) Moreover, multi-group analyses revealed differences in the IPI model among audiences with different attitudes toward the COVID-19 vaccine. These differences demonstrated that prior attitudes toward the COVID-19 vaccine can influence attitudes toward similar influenza vaccines, thus demonstrating the existence of spillover effects.

**Conclusion:**

Attitude toward the COVID-19 vaccine can influence the intention to receive the influenza vaccination. Those with a negative attitude toward the COVID-19 vaccine are significantly influenced by susceptibility to influenza. Perceived media influence affects the intention to receive the influenza vaccination among those with a positive attitude toward the COVID-19 vaccine.

## 1 Introduction

COVID-19 is a highly infectious disease caused by the novel coronavirus (SARS-CoV-2), and as of April 2023, 760 million confirmed cases have been reported globally, of which 6,887,000 have died (1). Its outbreaks have long disrupted people’s normal lives and inflicted devastating impacts on global health, safety, economy, and trade. Some studies have shown that vaccination is effective in preventing COVID-19. Statistics from the UK found that among adults aged 18–69 years, receiving two doses of the COVID-19 vaccine at least 2 weeks before the initial diagnosis reduced the infection rate by 41.1% (2). Although the COVID-19 pandemic is currently under control and the virus has become much less virulent, seasonal influenza remains a concern and apprehensions about it have not yet subsided. While many scholars have focused on COVID-19 vaccine hesitancy due to the devastating nature of the disease, the situation with influenza vaccination is equally discouraging. Influenza viruses are life-threatening respiratory pathogens for people with weakened immune systems. Vaccination is an important preventive measure against seasonal influenza, but currently, influenza vaccination coverage remains low in many countries (3).

It has been suggested that although COVID-19 and influenza are caused by different viruses, both are infectious respiratory illnesses. Therefore, a higher perceived risk of COVID-19 may lead to greater willingness to receive the seasonal influenza vaccine (4). There have been a few studies that have noted that attitudes toward the COVID-19 vaccine may influence attitudes toward the influenza vaccine and promote higher influenza vaccination rates. A study in Hong Kong found that COVID-19 may increase influenza vaccination rates to nearly 50% (5). For several seasons prior to the 2018–19 season, influenza vaccination coverage had remained the same for children (6). However, influenza vaccination coverage has changed following the COVID-19 outbreak. Notably, during the 2019–2020 season, both children and adults experienced an increase in influenza vaccination coverage compared to the preceding 2018–2019 season (7). In a survey of a certain region in China, changes in intention to receive influenza vaccination were also found. The influenza vaccination rate for the 2021–2022 season stood at 17.68%, marking a significant increase compared to the 11.8% rate observed in the 2018–2019 season. This upward trend was consistent across various age groups within the population (8). While the COVID-19 outbreak is currently under control and no longer considered a “public health emergency of international concern,” its impact persists. In particular, it remains unclear how vaccination against COVID-19 affects vaccination against influenza, a similar respiratory illness. Therefore, this paper asks: “How do pre-existing attitudes toward the COVID-19 vaccine affect intention to receive influenza vaccination?”

To investigate this question, this study first explored whether the influence of presumed influence model (IPI model) is valid in the context of influenza vaccination, based on the theory of the influence of presumed influence. Specifically, it examined how people’s exposure to the influenza vaccine information affects their own attitudes and intentions toward influenza vaccination through perceived media influence on others. The influence of presumed influence model posits that people adjust their attitudes and behaviors according to perceived media influence on others (9). This study conducted a multi-group analysis based on the theory of the influence of presumed influence model to investigate whether there are differences in the model between respondents with varying attitudes toward the COVID-19 vaccine. This examined the potential spillover effects of the COVID-19 vaccine on influenza vaccination. This study aimed to answer these questions in the context of both influenza vaccination and COVID-19 vaccination, although the COVID-19 outbreak and vaccination campaign are currently in the past. Concerns regarding the influenza vaccine remain prevalent, leading to cautious attitudes and behaviors toward this vaccine. It is hoped that exploring factors influencing vaccination may provide useful insights for future vaccination communication strategies.

## 2 Literature review

### 2.1 The influence of presumed influence model

One’s intention to vaccine could be influenced by information exposure to vaccines on social media, and reading comments expressing opinions on vaccines can lead to changes in one’s attitude toward vaccines, which could guide vaccination behavior (10, 11). Studies on the indirect influence of media have received increasing attention compared to the studies on the direct influence of media. One focus of this study is to examine how people’s attention to the influenza vaccine information in media indirectly influences their attitudes toward the influenza vaccine and intention to receive influenza vaccination. In this paper, the influence of presumed influence model proposed by Gunther was used to test how information exposure to the influenza vaccine indirectly affects their attitudes toward the influenza vaccine and intention to receive influenza vaccination.

Gunther first proposed the influence of presumed influence (IPI) model, which centers on the assumption that “people perceive the influence of media on others (presumed influence) and change their attitudes or behaviors as a result (influence of presumed influence)” [(12), p. 199]. The model posits that individuals infer others’ media information exposure based on their own exposure and the degree to which individuals believe they perceive media messages determines the degree to which others are influenced. Thus, whether or not an individual is influenced by a media message, they will make corresponding changes in attitude and behaviors through their perceived influence on others. Gunther’s study found that when adolescents were exposed to pro-smoking media messages, they perceived their peers to be more influenced, which, in turn, shaped their own intention to smoke (13). In other words, the IPI model produces behavioral outcomes, as individuals perceive media influences on others may alter personal attitudes and behaviors (9, 12).

The IPI model, starting with perceived influence on others stemming from individual exposure, has been demonstrated in domains such as healthy lifestyles, violent video games, and more (14, 15). According to the IPI model, information exposure to the influenza vaccine affects perceived media influence on others. For example, when people encounter pro-vaccine comments, they will think that others have been influenced by COVID-19 vaccine promotional posts and thus accepted the COVID-19 vaccine (16). Therefore, perceived media influence on others occurs when exposed to the influenza vaccine messages. Exposure is significantly correlated with perceived influence on others, leading to the hypothesis:

*H1:* Information exposure to the influenza vaccine significantly affects perceived media influence on others.

Perceived media influence on others can lead to subsequent changes, as people shift attitudes based on how exposure affects others. Studies have found that people’s perception of media influence on others can predict their own intention to engage in health behaviors, which has been validated across domains, including weight loss, safe sex practices, healthy eating, and skin cancer prevention (17, 18). In other words, information exposure to the influenza vaccine creates a perceived influence on others leading people to change their attitude toward the influenza vaccine. Some studies also suggest that perceived influence directly changes behavioral intention, even without direct effects (12) or concurrently with them (19). Therefore, this paper proposes the following hypotheses:

*H2:* Perceived media influence on others significantly affects intention to receive influenza vaccination.

*H3:* Perceived media influence on others significantly affects attitudes toward the influenza vaccine.

Studies have shown that when respondents perceive avian influenza news as influencing others, individuals are more likely to seek out avian influenza vaccine and related information (20). This study predicts that audiences’ attitudes toward the influenza vaccine will affect their intention to receive influenza vaccination. Based on the established relationship between attitude and intention, the current study hypothesizes:

*H4:* Attitude toward the influenza vaccine significantly affects intention to receive influenza vaccination.

### 2.2 Susceptibility to influenza

Moreover, perceived susceptibility is a crucial factor in health decision-making and preventive behaviors (21). In addition, this study also examines the effect of susceptibility on attitude toward the influenza vaccine and intention. According to the health belief model (HBM), research has found that susceptibility can all shape decisions to vaccinate against seasonal and pandemic flu (22).

Prior studies have explored the relationship between information exposure to disease can alter susceptibility. One study found static advertising positively associated with smoking susceptibility in adolescents, while movie/TV exposure was not significant (23). In COVID-19 research, social media information exposure positively correlated with susceptibility in pregnant women (24). However, some intergenerational studies find no direct relationship between media exposure and susceptibility (25). Based on these findings, this paper hypothesizes:

*H5:* Information exposure to the influenza vaccine will significantly increase susceptibility to influenza.

Influenza vaccination studies show high-risk, unvaccinated individuals believe prevention measures besides vaccines are effective, while those with lower perceived susceptibility have lower vaccination intentions (21). In the context of HPV vaccination, perceived susceptibility positively correlates with vaccination intention (26). A study of 300 female students found that increased susceptibility led to higher vaccination intention (27). A study found no direct, significant influence, perhaps due to optimism bias reducing perceived susceptibility (25). Despite some inconsistent findings, most studies observe a positive correlation between susceptibility to influenza and intention to receive influenza vaccination. Therefore, this paper also hypothesizes:

*H6:* Susceptibility to influenza will significantly increase the intention to receive influenza vaccination.

### 2.3 Spillover effects

A number of studies have verified the IPI model. Influenza is a contagious respiratory illness generally caused by influenza A and B viruses. However, characterized by antigenic variability, rapid spread, and annual seasonal epidemics, influenza results in approximately 1 billion infections and 300,000–500,000 deaths per year according to World Health Organization estimates (28). Vaccination is the most cost-effective prevention method; however, influenza vaccination coverages in China remain relatively low compared to other countries (29). Studies have confirmed vaccination policy, knowledge, and history are key factors influencing influenza vaccination (28–31). Meanwhile, vaccine misinformation online can negatively impact willingness (32).

Individuals’ perceptions of one familiar technology may spill over into their assessment of a new technology (33). Research shows perceptions of genetically modified foods influence views on nanofoods and nanotechnology labeling. If people see GMOs as beneficial, they are less supportive of nano labeling even if unconvinced of nanotechnology benefits (34). Numerous studies have focused on cross-vaccine effects, meaning attitudes toward one vaccine can influence views of similar vaccines. For example, past research found those vaccinated against influenza were more likely to receive pandemic vaccines (35).

While most cost-effective for public health, some vaccine hesitancy driven by safety concerns persists, being named a top 10 threat in 2019 by WHO. Studies show associations between influenza and COVID-19 vaccine intent. Higher perceived COVID-19 risk increases seasonal influenza vaccine intent given similar transmission and symptoms (4). A study in Italy found that regardless of people’s views on vaccines, intentions to the influenza vaccine increased with higher perceived risk. At the same time, research shows that people who have received the influenza vaccine are more willing to get the COVID-19 vaccine (36). While prior research has suggested some positive association between COVID-19 vaccine attitude and influenza vaccine uptake, the mechanism behind this correlation remains unclear. Therefore, this paper addresses the following question:

Q1: Do variations in attitudes toward the COVID-19 vaccine moderate the influence of the presumed influence (IPI) model?

**Figure 1:**
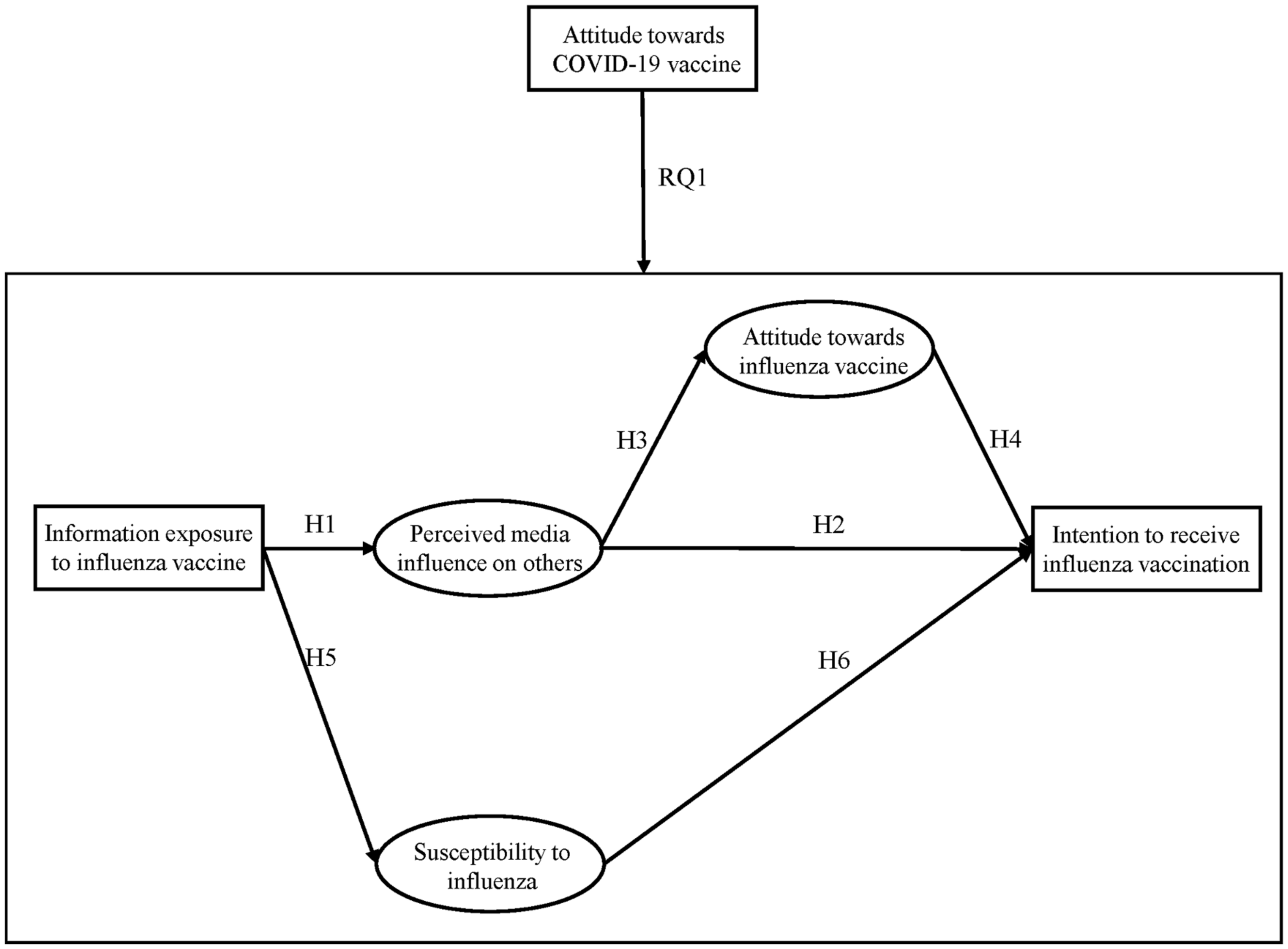
Model of this paper.

In conclusion, information exposure to the influenza vaccine is an important factor influencing audience attitudes toward the influenza vaccine and intention to receive influenza vaccination. Figure 1 outlines the assumptions and research questions examined in this paper. Accordingly, based on the IPI model, this paper specifically examines how exposure to the influenza vaccine information indirectly affects people’s intention to receive influenza vaccination, in other words, how people’s perceived media influence on others affects their intention. At the same time, this paper explores whether individuals’ attitudes toward the COVID-19 vaccine spill over into their attitudes toward the influenza vaccine.

## 3 Data and method

### 3.1 Data

This study employed a questionnaire survey targeting college students, conducted via the “Wenjuanxing” platform from 1 June to 7 June 2023. A total of 631 questionnaires were collected, and after excluding responses with logical conflicts, 613 questionnaires were deemed valid, resulting in a recovery rate of 97.15%.

### 3.2 Measures

#### 3.2.1 Information exposure to the influenza vaccine

Respondents answered the question about their information exposure to the influenza vaccine, which was adapted from Gunther and Storey (12). Data were collected using a 5-point Likert scale (1 = strongly disagree, 2 = disagree, 3 = fairly agree, 4 = agree, and 5 = strongly agree) for this question. Higher values on the scale indicated greater concern about information regarding the influenza vaccine.

#### 3.2.2 Perceived media influence on others

Respondents answered 3 perceived influences of the influenza vaccine on others scenarios, with reference groups including family members, friends, and others, with higher scores indicating that self-perceived influenza vaccine messages have a greater influence on others. This study used the Perceived Media Influence on Others Scale (PMIO), adapted from Paek and Gunther (37, 38). The data were collected using a 5-point Likert scale (1 = very small, 5 = very large) for this question.

#### 3.2.3 Susceptibility to influenza

Individuals have different subjective perceptions when dealing with different diseases. Susceptibility to influenza is a term commonly used in the medical literature, and this paper used the Susceptibility to Influenza Scale (SI), referred to the paper (39). Measuring the susceptibility to influenza: “I am susceptible to colds, influenza, and other infectious diseases”, “I think I am at a higher risk of getting the influenza than the people around me”, “If I get the influenza”, “I will not be able to do my daily activities”, and “I worry that the influenza will make me very sick”, and scores were calculated using a 5-point Likert scale. Higher scores indicated higher perceived illness.

#### 3.2.4 Attitude toward the influenza vaccine

This paper measures attitudes toward the influenza vaccine and COVID-19 vaccine using the same set of items, which used Attitude toward the Influenza Vaccine Scale (AIV), adapted from Martin’s study (40). After testing the scale for high internal consistency and reliability, a series of adaptations were made to measure attitudes toward the influenza vaccine separately, taking into account the national context and reality. Attitudes toward the influenza vaccine were measured using three questions: “Getting influenza vaccine makes me feel safe”, “I can rely on the influenza vaccine to stop me being attacked by the influenza”, and “Getting influenza vaccine makes me feel protected”. The question was used to collect data on a 5-point Likert scale (1 = strongly disagree, 2 = disagree, 3 = fairly agree, 4 = agree, and 5 = strongly agree), with larger values indicating more positive attitudes toward the influenza vaccine.

#### 3.2.5 Intention to receive influenza vaccination

In this paper, the same Likert 5-point scale was used to collect data on the intention to receive influenza vaccination (4): “I am willing to receive influenza vaccination” (1 = very unwilling, 5 = very willing).

Finally, demographic factors and seven demographic variables were used as control variables in this study: gender, age, nation, place of origin, major, grade, and monthly consumption level. The specific coding for these variables is as follows: “gender” (male = 1; female = 0), “nation” (Han Chinese = 1; minority = 0), “major” (natural science = 1; humanities and social science = 0), “place of origin” (eastern region = 1; midwestern region = 0), and “grade” (freshmen = 1; non-freshmen = 0). Age was measured as a continuous variable. The monthly consumption level was collected by asking respondents to indicate their consumption interval, after which the midpoint of the interval was calculated and divided by 1,000 to represent the monthly consumption level.

Based on the compilation and generalization of related research literature, this study identified questions to measure the variables and revised them to take into account the national context. These revised questions were then validated through pre-survey tests, and the results met the reliability and validity requirements.

### 3.3 Analytical approach

Data analysis was performed using MPlus for structural equation modeling (SEM). To account for the non-normal data distribution, the analysis employed a robust maximum-likelihood estimator (MLMV). The model included gender, age, nation, place of origin, major, grade, and monthly consumption level as control variables.

Model fit was evaluated based on the following criteria: (a) an insignificant maximum-likelihood chi-square ( $\chi^{2}$ ) value ( p>0.05 ); (b) a relative chi-square ratio ( $\chi^{2}/df$ ) less than 5 (41); (c) a root mean square error of approximation (RMSEA) below 0.08 (42); (d) a standardized root mean square residual (SRMR) below 0.08 (43); and (e) comparative fit index (CFI) and Tucker–Lewis index (TLI) exceeding 0.95 (44).

To answer the research questions posed in this paper, and to test whether there is a difference in the IPI model between those with different attitudes toward the COVID-19 vaccine, participants were categorized into two groups: those with negative attitudes ( $M_{\mathrm{attitude}}$ ≤3.0) and those with positive attitudes ( $M_{\mathrm{attitude}}$ >3.0). In particular, 20.4% of respondents had negative attitudes. Multi-group comparative analyses were conducted to test for differences between these two groups. First, a multi-group model was run for two groups—those with negative attitudes toward the COVID-19 vaccine and those with positive attitudes toward the COVID-19 vaccine. Following this, a similar multi-group model was run with all path coefficients constrained to be equal. Using the DIFFTEST command in Mplus, a chi-square difference test was performed to compare the unconstrained and constrained multi-group models. For statistically different groups, the chi-square difference test should produce significant changes (45). Subsequently, a series of multi-group models were conducted, which contained only specific path coefficients constrained to be equal. These constrained multi-group models were compared to unconstrained multi-group models by performing a series of chi-square difference tests to check whether the differences were located on specific individual paths within the models.

## 4 Results

### 4.1 Descriptive statistics

This paper mainly focuses on the college student group. Of the 613 respondents, 45.4% ( n=278 ) were men and 54.6% ( n=335 ) were women, which is consistent with the gender ratio of the college student population. The age ranged from 17 to 35 years with a median of 22 years, matching the profile of college students. In terms of geographic distribution, 47% were from eastern provinces and 53% from central and western provinces. Ethnically, 92.8% were Han Chinese, reflecting the ethnic composition. Regarding academic majors, 47.8% were in natural sciences and 52.2% in humanities and social sciences. Freshmen accounted for 23.7% of the sample. The mean monthly consumption level was 2,400 yuan. In summary, the sample characteristics, including gender, age, nation, place, major, grade, and monthly consumption level, are largely representative of the college student population (see Table 1).

**Table 1:** Descriptive statistics.

Variables	N (percentage) or M(SD)
Information exposure to the influenza vaccine	2.93 (1.01)
Intention to receive influenza vaccination	4.00 (0.86)
Gender	
Male	278 (45.4%)
Female	335 (54.6%)
Age	22.00 (2.42)
Nation	
Han	569 (92.8%)
Minority	44 (7.2%)
Place	
East	288 (47.0%)
Central and west	325 (53.0%)
Fresh	
Fresh	146 (23.8%)
Non-freshmen	467 (76.2%)
Monthly consumption level	2.40 (3.14)
Major	
Natural sciences	293 (47.8%)
Humanities & social sciences	320 (52.2%)

### 4.2 Measurement models

To examine the reliability of the indicators (attitude toward the influenza vaccine, perceived media influence on others, and susceptibility to influenza), Cronbach’s α and composite reliability (CR) coefficients were utilized in this study. Factor loadings above 0.5, Cronbach’s alpha above 0.7, and CR above 0.6 suggested adequate internal consistency. Moreover, average variance extracted (AVE) was inspected for each latent variable to ensure the items sufficiently contributed to the intended constructs, with values above 0.5 deemed satisfactory. As shown in Table 2, the findings supported the reliability of the measures used to address the research questions.

**Table 2:** Results of the confirmatory factor analysis.

Construct	Item	Standardized factor loading	Cronbach’s α	CR	AVE
Attitude toward the influenza vaccine	FA1	0.794	0.827	0.835	0.629
	FA2	0.707			
	FA3	0.870			
Perceived media influence on others	FI1	0.861	0.840	0.843	0.644
	FI2	0.850			
	FI3	0.684			
Susceptibility to influenza	FS1	0.859	0.839	0.836	0.567
	FS2	0.862			
	FS3	0.637			
	FS4	0.616			

CR, composite reliability; AVE, average variance extracted; FA1, “I feel safe after being vaccinated”; FA2, “I can rely on vaccine to stop serious infectious diseases”; FA3, “I feel protected after getting vaccinated”; FI1, “Perceived media influence of influenza vaccine information on my family”; FI2, “Perceived media influence of influenza vaccine information on my friends”; FI3, “Perceived media influence of influenza vaccine information on others”; FS1, “I get colds, influenza, and other contagious diseases very easily”; FS2, “I think I’m at a higher risk for influenza than those around I think I’m at a higher risk for influenza than those around me”; FS3, “I had influenza and I could not do my daily activities”; FS4, “I’m worried that influenza will be a problem for me”.

As shown in Table 3, these models’ fit indices indicated adequate fit.

**Table 3:** Fit indices for measured and structural models.

	Measurement model	Structural model
$\chi^{2}/df$	2.715	3.620
RMSEA	0.053	0.065
SRMR	0.036	0.062
CFI	0.981	0.918
TLI	0.972	0.882

### 4.3 Hypothesis testing

#### 4.3.1 Validation of the IPI model results

The model fit for this paper was good (Table 3), indicating the model was supported by the data. The results showed a positive correlation between respondents’ information exposure to the influenza vaccine and perceived media influence on others ( β=0.365 , p<0.001 ). This correlation was statistically significant, confirming H1.

Hypothesis 3 proposed a significant association between perceived media influence on others and attitudes toward the influenza vaccine. The results revealed a positive correlation between these two variables ( β=0.159 , p<0.01 ), providing statistical support for H3.

Hypothesis 4 proposed that attitude toward the influenza vaccine significantly influences the intention to receive influenza vaccination. The analysis uncovered a positive correlation between these variables ( β=0.0649 , p<0.001 ), confirming this hypothesis.

Hypothesis 5 states that exposure to vaccine information relates to susceptibility to influenza. The data revealed a positive association ( β=0.242 , p<0.001 ), supporting H5.

Hypothesis 6 proposed an association between susceptibility and intention to receive influenza vaccination. Results showed a positive correlation ( β=0.097 , p<0.05 ), confirming this hypothesis.

Perceived media influence did not directly relate to the intention to receive influenza vaccination ( β=0.012 , p>0.05 ). Thus, H2 was not supported.

Regarding controls, gender, age, nation, and origin place impacted attitude and susceptibility. However, monthly consumption level, major, and grade did not relate to vaccine intention.

**Figure 2:**
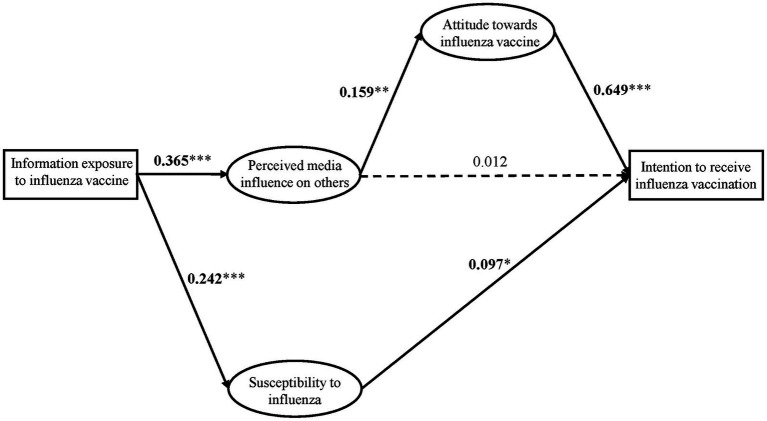
Results of the structural model. The coefficients in the graphs are standardized beta coefficients; dashed lines indicate unconfirmed; and solid lines indicate confirmed. * p<0.05 ; ** p<0.01 ; *** p<0.001 is reflective of significance.

In summary, the model was supported, confirming all hypotheses except H2. Figure 2 visualizes these results.

#### 4.3.2 Validation of the results of multi-group analysis

To examine whether there were differences in the IPI model between those with positive and negative attitudes toward the COVID-19 vaccine, a multi-group analysis was conducted. The results of the chi-square test of variance showed a significant between-group difference between the two models ( $\Delta \chi^{2}\left(6\right)=26.238$ , p<0.001 ), indicating the IPI model varied based on attitude toward the COVID-19 vaccine.

A series of follow-up chi-square variance tests revealed significant between-group differences in three specific model paths. First, there was a difference between attitude toward the influenza vaccine and intention to receive influenza vaccination ( $\Delta \chi^{2}\left(1\right)=6.510$ , p<0.05 ). Second, a difference existed between susceptibility to influenza and intention to receive influenza vaccination ( $\Delta \chi^{2}\left(1\right)=7.208$ , p<0.01 ). Finally, a difference was found between perceived media influence on others and attitudes toward the influenza vaccine (∆ $\chi^{2}$ (1) = 7.490, p<0.01 ). Taken together, these results demonstrate that attitude toward the COVID-19 vaccine moderated the relationships between attitude toward the influenza vaccine and intention to receive influenza vaccination, susceptibility to influenza and intention to receive influenza vaccination, and information exposure to the influenza vaccine and attitude toward the influenza vaccine (see Table 4).

**Table 4:** Multi-group analysis.

Multi-group models	$\chi^{2}$	*df*	$\chi^{2}/df$	CFI	TLI	RMSEA	SRMR
Unconstrained	484.143	212	2.284	0.886	0.847	0.065	0.066
Constrained (all paths)	509.904	218	2.339	0.878	0.840	0.066	0.083
Chi-square difference test	$\Delta \chi^{2}\;\left(6\right)=26.238$ , p<0.001						
Unconstrained	484.143	212	2.284	0.886	0.847	0.065	0.066
Constrained (attitude intention)	490.462	213	2.303	0.884	0.845	0.065	0.068
Chi-square difference test	$\Delta \chi^{2}\;\left(1\right)=6.510\text{,}$ p<0.05						
Unconstrained	484.143	212	2.284	0.886	0.847	0.065	0.066
Constrained (susceptibility intention)	491.188	213	2.306	0.884	0.844	0.065	0.070
Chi-square difference test	$\Delta \chi^{2}\;\left(1\right)=7.208$ , p<0.01						
Unconstrained	484.143	212	2.284	0.886	0.847	0.065	0.066
Constrained (perceived media influence on others—intention)	484.718	213	2.276	0.887	0.848	0.065	0.066
Chi-square difference test	$\Delta \chi^{2}\;\left(1\right)=0.178$ , p>0.05						
Unconstrained	484.143	212	2.284	0.886	0.847	0.065	0.066
Constrained (perceived media influence on others—attitude)	490.426	213	2.302	0.884	0.845	0.065	0.071
Chi-square difference test	$\Delta \chi^{2}\;\left(1\right)=7.490$ , p<0.01						
Unconstrained	484.143	212	2.284	0.886	0.847	0.065	0.066
Constrained (information exposure to the influenza vaccine—perceived media influence on others)	486.268	213	2.283	0.886	0.847	0.065	0.068
Chi-square difference test	$\Delta \chi^{2}\;\left(1\right)=2.441$ , p>0.05						
Unconstrained	484.143	212	2.284	0.886	0.847	0.065	0.066
Constrained (information exposure to the influenza vaccine—susceptibility)	484.740	213	2.276	0.887	0.848	0.065	0.067
Chi-square difference test	$\Delta \chi^{2}\;\left(1\right)=0.471\text{,}$ p>0.05						

**Figure 3:**
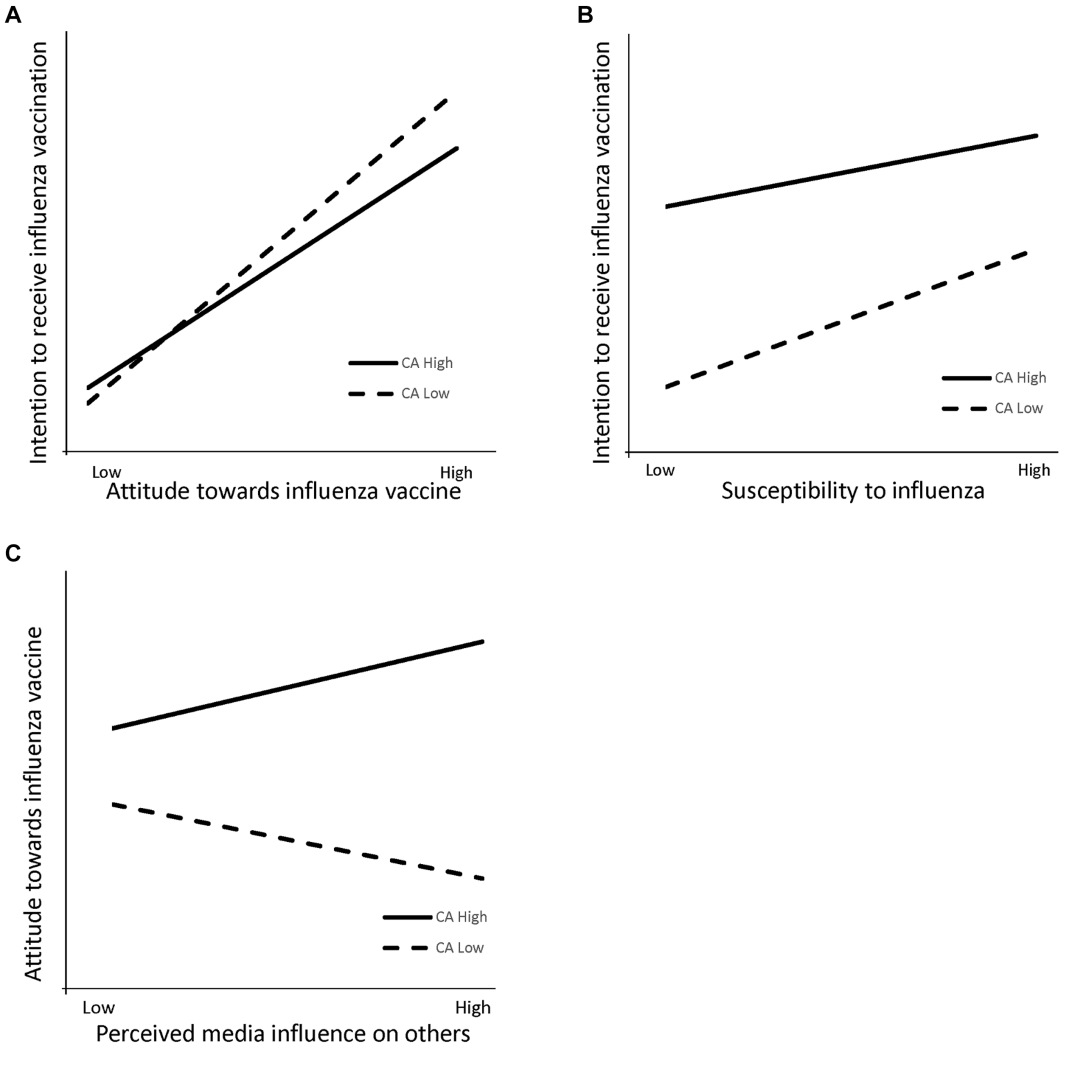
**(A)** Effect of interaction between attitude toward influenza vaccine and attitude toward COVID-19 vaccine on intention to receive influenza vaccination. **(B)** Effect of interaction between susceptibility to influenza and attitude toward COVID-19 vaccine on intention to receive influenza vaccination. **(C)** Effect of interaction between perceived media influence on others and attitude toward the COVID-19 vaccine on attitude toward the influenza vaccine. CA = Attitude toward the COVID-19 vaccine.

Specifically, attitude toward the influenza vaccine significantly predicted intention to receive influenza vaccination in both the positive ( β=0.776 , p<0.001 ) and negative ( β=1.010 , p<0.001 ) COVID-19 vaccine attitude groups, although the strength differed (Figure 3A). Additionally, susceptibility to influenza only was associated with the intention for those negative attitudes toward the COVID-19 vaccine ( β=0.130 , p<0.05 ), implying increasing susceptibility may raise the intention to receive influenza vaccination in this group (Figure 3B). Finally, perceived media influence was related to attitude toward the influenza vaccine for those positive attitudes toward COVID-19 vaccine ( β=0.117 , p<0.01 ), but not for those negative ( β=−0.100 , p>0.05 ), suggesting perceived media influence on others does not directly affect attitude to the influenza vaccine among this group (Figure 3C). In summary, this multi-group analysis showed that attitude toward the COVID-19 vaccine significantly impacted the IPI model pertaining to influenza vaccination, answering the core research question.

## 5 Discussion

This study analyzed the IPI model on influenza vaccination intention using an online questionnaire grounded in the frameworks of the IPI model and spillover effects. The results showed all hypotheses were supported except for the non-significant relationship between perceived media influence on others and intention to receive influenza vaccination ( β=0.012 , p>0.05 ). A multi-group analysis based on spillover effects tested models’ differences between those with positive and negative attitudes toward the COVID-19 vaccine. Significant between-group differences emerged for three pathways: (1) the path from attitude toward the influenza vaccine to intention to receive influenza vaccination ( $\Delta \chi^{2}\;\left(1\right)=6.510$ , p<0.05 ); (2) the path from susceptibility to influenza to intention to receive influenza vaccination ( $\Delta \chi^{2}\;\left(1\right)=7.208$ , p<0.01 ); and (3) the path from perceived media influence on others to attitude toward the influenza vaccine ( $\Delta \chi^{2}\;\left(1\right)=7.490$ , p<0.01 ).

Overall, the IPI model is still valid in the context of the intention to receive influenza vaccination. Greater information exposure to the influenza vaccine increased perceived media influence on others. In turn, the higher perceived influence was related to a more positive attitude toward the influenza vaccine and a greater intention to receive influenza vaccination. Additionally, information exposure to the influenza vaccine was associated with higher susceptibility to influenza, which also predicted intention to receive influenza vaccination. In particular, perceived media influence on others did not directly impact intention to receive influenza vaccination, indicating wariness about the vaccine. Attitudes may need to shift before intention strengthens this critical health behavior. In summary, while perceived social influence alone did not motivate intention to receive influenza vaccination, changing attitudes and overcoming barriers may generate intention to receive influenza vaccination.

This study also conducted a multi-group analysis based on spillover effects to examine models’ differences between those with positive or negative attitudes toward the COVID-19 vaccine. The results revealed significant differences in the IPI model between the two vaccine attitudes groups, demonstrating a “spillover effect.” This aligns with past research showing individuals’ attitudes toward one vaccine can influence their attitudes toward a similar vaccine, impacting vaccination behavior (46, 47). The further multi-group analysis uncovered spillover effects for three pathways: attitude toward the influenza vaccine and intention to receive influenza vaccination; susceptibility to influenza and intention to receive influenza vaccination; and perceived media influence on others and attitude toward the influenza vaccine. This shows a positive relationship between attitude toward the influenza vaccine and intention to receive influenza vaccination. However, the strength of this influence can vary depending on their attitudes toward the COVID-19 vaccine. The effect was stronger for those with negative attitudes toward the COVID-19 vaccine. In the context of positive attitudes toward the COVID-19 vaccine, the greater the perceived media influence on others and the greater susceptibility to influenza, making them more likely to want to get the influenza vaccine themselves. In the case of negative attitudes toward the COVID-19 vaccine, the higher the susceptibility to influenza, the more likely intention to receive influenza vaccination. The greater the perceived media influence on others, the lower the intention to receive influenza vaccination. These findings highlight that attitude toward the influenza vaccine remains the most important factor for intention to receive influenza vaccination, but the strength of this association can be modulated by attitude toward COVID-19 vaccine and susceptibility to influenza. The multi-group analysis successfully answered the research questions, revealing spillover effects between COVID-19 and attitude toward the influenza vaccine and intention to receive influenza vaccination.

In summary, the multi-group analysis provided important insights into the spillover effects between influenza and COVID-19 vaccine attitudes. Overall, influenza vaccine attitude significantly and positively predicted intention to receive influenza vaccination regardless of COVID-19 vaccine attitude. However, susceptibility only influenced intention among those negative attitudes toward the COVID-19 vaccine. This implies increasing perceived susceptibility could improve the intention to vaccinate against influenza in this group. Additionally, perceived media influence on others was related to more positive attitudes toward the influenza vaccine and, in turn, greater intention to receive influenza vaccination, but only for those positive attitudes toward the COVID-19 vaccine. Therefore, the multi-group analysis demonstrated pre-existing spillover effects between vaccine attitudes, though the pathways differed based on COVID-19 vaccine attitudes. For those negative attitudes toward the COVID-19 vaccine, influencing susceptibility to influenza could improve the influenza vaccine uptake. However, for those positive attitudes toward the COVID-19 vaccine, leveraging media influence to shape attitudes may increase the intention to receive the influenza vaccine. In conclusion, accounting for COVID-19 vaccine spillover effects allows tailored targeting of the critical factors shaping influenza vaccination for each attitude group.

### 5.1 Limitations

Of course, there are some limitations of the research presented in this paper. First, the survey was primarily conducted online, which may introduce selection bias in the sample. Although efforts were made to maximize sample diversity during recruitment, the randomness of the sample cannot be guaranteed. As such, the results may not fully generalize to the broader population. Future research should aim to achieve a more representative sample through diverse recruitment strategies and should include populations from different geographical, socioeconomic, and cultural backgrounds. Additionally, the survey captured attitude at a single point in time. We cannot elucidate how attitudes shift in response to changes in virus risk or vaccine availability. Future research should further examine whether spillover effects from domains outside of vaccination significantly influence the IPI model. Other limitations include the reliance on self-reported survey measures, which can be prone to biases like social desirability. The cross-sectional nature of the data also precludes determining causality. Longitudinal or experimental data could provide stronger evidence for causal relationships between the assessed factors over time. Overall, while this study provides meaningful insights into spillover effects on vaccine attitude, the limitations highlight important avenues for future research. Further studies with more representative, diverse samples, and multiple timepoints could build upon these findings.

### 5.2 Implications for research and practice

This study makes several key contributions to the literature. First, it demonstrates the applicability of the IPI model in the vaccine context, providing theoretical backing for factors shaping attitudes toward the influenza vaccine and intention to receive influenza vaccination. More importantly, it reveals prior vaccine attitude can impact the model for related vaccines—there are group differences based on COVID-19 vaccine attitude. For those negative attitudes toward the COVID-19 vaccine, susceptibility to influenza strongly was associated with the intention to receive influenza vaccination, while media influence was related to more positive influenza attitudes for those positive attitudes toward the COVID-19 vaccine. These findings allow more targeted communication to different attitude groups to optimize vaccine uptake. Though the COVID-19 pandemic has passed, vaccine hesitancy persists for other public health vaccines like influenza. While the specific virus changes, vaccine reluctance itself remains an obstacle to promoting population health. By elucidating the pathways shaping the influenza vaccine attitude and intent among different COVID-19 vaccine attitude groups, this study provides valuable insights to inform targeted, effective health communication to improve vaccine acceptance. Overall, accounting for spillover effects between vaccine attitudes allows tailored messaging to address the key drivers of vaccine hesitancy for each group.

## 6 Conclusion

This study has found that attitude toward the COVID-19 vaccine can impact the intention to receive influenza vaccination. Specifically, susceptibility to influenza affects individuals who hold a negative attitude toward the COVID-19 vaccine. On the other hand, for individuals who hold positive attitudes toward the COVID-19 vaccine, perceived media influence on others affects their intention to receive influenza vaccination. This paper aims to explore the impact of information exposure to the influenza vaccine on attitude and intention toward influenza vaccination, with the hope of providing insights for vaccine promotion strategies and fostering the development of health communication.

## Data Availability

The raw data supporting the conclusions of this article will be made available by the authors, without undue reservation.
